# Supplementary material for: Learning curve and surgical outcome of robotic assisted colorectal surgery with ERAS program
Source: Sci Rep. 2022 Nov 29;12:20566. doi: 10.1038/s41598-022-24665-w (PMC9709162; doi:10.1038/s41598-022-24665-w)
Supplement: Supplementary file 1 — Supplementary Information 1. [file 41598_2022_24665_MOESM1_ESM.zip › LSC Raw data-Ñ[▒K/131/O_ñw╝╨░O▒Kñσ.pdf]

正式摘要(電子簽章：000000222914981)

W95 / W95

臺中榮民總醫院

# Operation Note

[redacted]

- 手術日期：2021/08/11
- 手術醫師：VS 林俊余，CR 陳映菁，R 郭連雄
- Anesthesia：GE
- Position：Supine
- Aseptic agents：Soap，Betadine Alcohol，Betadine Aqua

## Diagnosis

- Pre-operative diagnosis :Chronic abdominal pain with proximal A-colon diverticulitis
- Post-operative diagnosis : Ditto.

## Surgical Indication

- symptom relief for chronic abdominal pain

## Operation

- 1. Laparoscopic right hemicolectomy with side-to-side anastomosis, endo GIA 60-3.8mm*2+endo GIA 60-4.1mm*1 and Connel suture with 3-0 monocryl. re-enforced with 3-0 mersilk lembert suture.
  2. Vessel ligation: ileo-colic vessels.
  3. A Fr.12 penrose drain placed at Morrison's pouch.

## Finding

- 1. multiple diverticulosis over A-colon
  2. No synchronous polyp or tumor at resected specimen.
  3. Mild adhesion at RLQ

## Procedure

- General endotracheal anesthesia was induced and then the patient was placed in the supine position with legs in adducted position. Preoperative antibiotics were given. A Foley catheter and nasogastric tube were placed. The abdomen was prepped and draped in standard sterile fashion. A small vertical incision was made above the umbilicus and a 12mm trocar was inserted into the peritoneal cavity by open method. Pneumoperitoneum was established with CO2 inflation. Laparoscope was inserted and then a 12mm trocar was inserted over LUQ of abdomen. Two 5mm trocars were inserted over RLQ and LLQ of abdomen. The small bowel was inspected and retracted to downward by grasper. The peritoneum overlying the mesentery was then scored with electrocautery and the ileocolic artery was identified, ligated with endoclips, and transected. The remaining vessels to the cecum were also divided and ligated. Using electrocautery, omentum was freed from the transverse colon and lesser sac was entered. The colon was freed from its peritoneal attachments along the line of Toldt from the cecum to the hepatic flexure. Mesentary of termial ileum was ligated. Hemostasis was checked in the operative field. Cecum, ascending colon and hepatic flexure were mobilized and then laparoscope was removed. A small midline incision was made over upper abdomen. The ascending colon was delivered into the incision and points of transection were selected proximally and distally. The bowel was divided. The two ends of the bowel were checked and found to be viable, with excellent blood supply. The fat was gently cleared from the terminal 2-3 mm of the bowel ends. The two ends of the bowel were brought into apposition and found to lie comfortably without excessive tension. endo GIA 60-4.1mm*1 was used to transect proximal T-colon, an endo GIA 60-3.8*1 was applied terminal ileum transection. engo GIA 60-3.8mm*1 wasapplied for common channel of T-colon and terminal ileum side-to-side anastomosis. Intestinal common channel orifice was sutured with Connel suture by 3-0 monocryl, an outer layer of

- interrupted 3-0 silk Lambert sutures was used for re-enforcement. The anastomosis was checked and found to be intact and widely patent. Then the anastomosis was put back into peritoneal cavity, and pneumoperitoneum was created again. We checked bleeding meticulously and penrose drain was placed at Morrison pouch. Drainage tube was inserted from LUQ 12mm trocar hole to Morrison pouch. The fascia of lower midline wound was closed with interrupted sutures of No.1 Vicryl.Skin were closed with interrupted 3-0 Nylon. The patient tolerated the procedure well and was brought to the POR unit in stable condition.
- Estimated blood loss: minimum
- 器械數量: 正確
- 紗布數量: 正確

## Image

- PAINT
  - abdomen

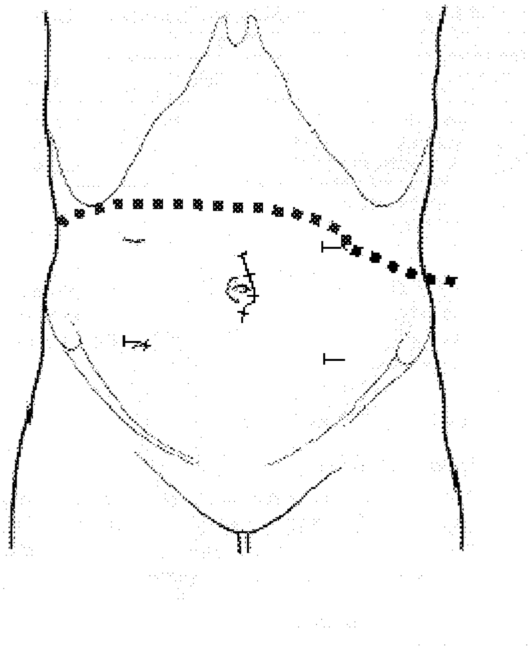

  - colon & rectum

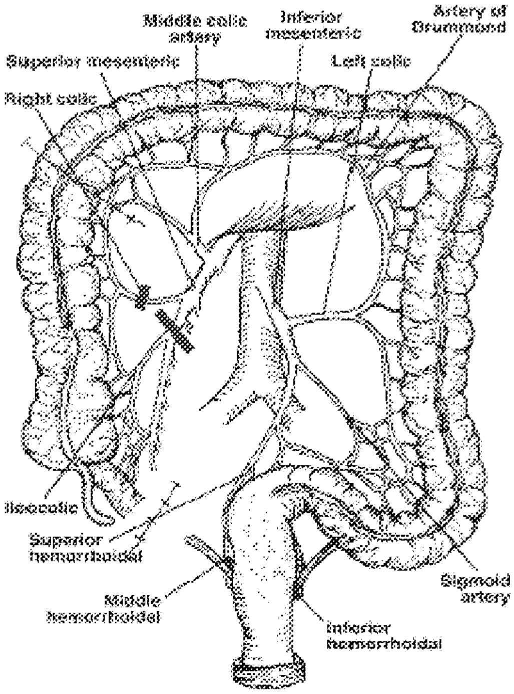


主治 2222H林俊余 / 醫師 2303K陳映菁

正式摘要(電子簽章：**000000222914981)**

W95 / W95

doctorRank:B
